# Injectables and implants in consideration of opioid use disorder treatment: Patient perspectives from India

**DOI:** 10.1192/j.eurpsy.2025.992

**Published:** 2025-08-26

**Authors:** S. Sarkar, N. Seth, M. K. Brar, A. Verma, S. Singh, A. Dhawan

**Affiliations:** 1 National Drug Dependence Treatment Centre; 2 Department of Psychiatry, All India Institute of Medical Sciences, New Delhi, India

## Abstract

**Introduction:**

Implants and injectable medications have been utilized frequently in the treatment of opioid use disorder, along with other treatment approaches like orally consumed opioid agonist treatment or antagonist treatment, and psychotherapeutic interventions. The overall use of implants and injectable medications in the Indian healthcare landscape is low.

**Objectives:**

The study aimed to assess the acceptability of implants and injectable among treatment options among patients with opioid use disorders.

**Methods:**

A cross sectional survey was conducted among patients with opioid use disorders. They were shown a pre-recorded video explaining the various treatment options for opioid use disorder, and their acceptability of treatment was recorded.

**Results:**

Among the 150 included patients, 26 (17.3%) were willing for naltrexone depots, 11 (7.3%) were willing for naltrexone implants, and 10 (6.7%) were willing for buprenorphine injection. Compared to this, patients were more willing for buprenorphine sublingual (n = 105, 70%), tramadol oral (n = 89, 59.3%), and naltrexone oral (n = 43, 28.7%) formulations. Willingness for counselling or psychotherapy was expressed by 34 patients (22.7%), and for engagement with narcotics anonymous by 20 patients (13.3%).

**Conclusions:**

While implants and injectable may be a viable option for many patients with opioid use disorder, it may have limited acceptability among the range of therapeutic options available. Collaborative decision making may be useful considering patient’s preferences for aligning goals of treatment team and the patient.

**Disclosure of Interest:**

None Declared

